# Computing the family-free DCJ similarity

**DOI:** 10.1186/s12859-018-2130-5

**Published:** 2018-05-08

**Authors:** Diego P. Rubert, Edna A. Hoshino, Marília D. V. Braga, Jens Stoye, Fábio V. Martinez

**Affiliations:** 1 Faculdade de Computação, Universidade Federal de Mato Grosso do Sul, Campo Grande, MS Brazil; 2 Faculty of Technology and Center for Biotechnology (CeBiTec), Bielefeld University, Bielefeld, Germany

**Keywords:** Genome rearrangement, Double-cut-and-join, Family-free genomic similarity

## Abstract

**Background:**

The genomic similarity is a large-scale measure for comparing two given genomes. In this work we study the (NP-hard) problem of computing the genomic similarity under the DCJ model in a setting that does not assume that the genes of the compared genomes are grouped into gene families. This problem is called family-free DCJ similarity.

**Results:**

We propose an exact ILP algorithm to solve the family-free DCJ similarity problem, then we show its APX-hardness and present four combinatorial heuristics with computational experiments comparing their results to the ILP.

**Conclusions:**

We show that the family-free DCJ similarity can be computed in reasonable time, although for larger genomes it is necessary to resort to heuristics. This provides a basis for further studies on the applicability and model refinement of family-free whole genome similarity measures.

**Electronic supplementary material:**

The online version of this article (10.1186/s12859-018-2130-5) contains supplementary material, which is available to authorized users.

## Background

A central question in comparative genomics is the elucidation of similarities and differences between genomes. Local and global measures can be employed. A popular set of global measures is based on the number of genome rearrangements necessary to transform one genome into another one [1]. Genome rearrangements are large scale mutations, changing the number of chromosomes and/or the positions and orientations of DNA segments. Examples of such rearrangements are inversions, translocations, fusions, and fissions.

As a first step before such a comparison can be performed, some preprocessing is required. The most common method, adopted for about 20 years [1, 2], is to base the analysis on the order of conserved syntenic DNA segments across different genomes and group homologous segments into *families*. This setting is said to be *family-based*. Without duplicate segments, i.e., with the additional restriction that at most one representative of each family occurs in any genome, several polynomial time algorithms have been proposed to compute genomic distances and similarities [3–7]. However, when duplicates are allowed, problems become more intricate and many presented approaches are NP-hard [2, 8–13].

Although family information can be obtained by accessing public databases or by direct computing, data can be incorrect, and inaccurate families could be providing support to erroneous assumptions of homology between segments [14]. Thus, it is not always possible to classify each segment unambiguously into a single family, and an alternative to the family-based setting was proposed recently [15]. It consists of studying genome rearrangements without prior family assignment, by directly accessing the pairwise similarities between DNA segments of the compared genomes. This approach is said to be *family-free* (FF).

The *double cut and join* (DCJ) operation, that consists of cutting a genome in two distinct positions and joining the four resultant open ends in a different way, subsumes most large-scale rearrangements that modify genomes [5]. In this work we are interested in the problem of computing the overall similarity of two given genomes in a family-free setting under the DCJ model. This problem is called FFDCJ similarity, and in some contexts it may be more powerful than a distance measure, where it is known that the parsimony assumption holds only for closely related genomes [16], while a well-designed similarity measure may allow more flexibility. As shown in [17], the complexity of computing the FFDCJ similarity was proven to be NP-hard, while the FFDCJ distance was already proven to be APX-hard. In the remainder of this paper, after preliminaries and a formal definition of the FFDCJ similarity problem, we first present an exact ILP algorithm to solve it. We then show the APX-hardness of the FFDCJ similarity problem and present four combinatorial heuristics, with computational experiments comparing their results to the ILP for datasets simulated by a framework for genome evolution.

A preliminary version of this paper appeared in the Proceedings of the 15th RECOMB Satellite Workshop on Comparative Genomics (RECOMB-CG 2017) [18].

## Methods

Each segment (often called *gene*) *g* of a genome is an oriented DNA fragment and its two distinct *extremities* are called *tail* and *head*, denoted by *g*^*t*^ and *g*^*h*^, respectively. A genome is composed of a set of chromosomes, each of which can be circular or linear and is a sequence of genes. Each one of the two extremities of a linear chromosome is called a *telomere*, represented by the symbol ∘. An *adjacency* in a chromosome is then either the extremity of a gene that is adjacent to a telomere, or a pair of consecutive gene extremities. As an example, observe that the adjacencies 5^*h*^, 5^*t*^2^*t*^, 2^*h*^4^*t*^, 4^*h*^3^*t*^, 3^*h*^6^*t*^, 6^*h*^1^*h*^ and 1^*t*^ can define a linear chromosome. Another representation of the same linear chromosome, flanked by parentheses for the sake of clarity, would be (∘ −5 2 4 3 6 −1 ∘), in which the genes preceded by the minus sign (−) have reverse orientation.

A *double cut and join* or DCJ operation applied to a genome *A* is the operation that cuts two adjacencies of *A* and joins the separated extremities in a different way, creating two new adjacencies. For example, a DCJ acting on two adjacencies *pq* and *rs* would create either the adjacencies *pr* and *qs*, or the adjacencies *ps* and *qr* (this could correspond to an inversion, a reciprocal translocation between two linear chromosomes, a fusion of two circular chromosomes, or an excision of a circular chromosome). In the same way, a DCJ acting on two adjacencies *pq* and *r* would create either *pr* and *q*, or *p* and *qr* (in this case, the operation could correspond to an inversion, a translocation, or a fusion of a circular and a linear chromosome). For the cases described so far we can notice that for each pair of cuts there are two possibilities of joining. There are two special cases of a DCJ operation, in which there is only one possibility of joining. The first is a DCJ acting on two adjacencies *p* and *q*, that would create only one new adjacency *pq* (that could represent a circularization of one or a fusion of two linear chromosomes). Conversely, a DCJ can act on only one adjacency *pq* and create the two adjacencies *p* and *q* (representing a linearization of a circular or a fission of a linear chromosome).

In the remainder of this section we extend the notation introduced in [17]. In general we consider the comparison of two distinct genomes, that will be denoted by *A* and *B*. Respectively, we denote by $\mathcal {A}$ the set of genes in genome *A*, and by $\mathcal {B}$ the set of genes in genome *B*.

### Adjacency graph and family-based DCJ similarity

**Fig. 1:**
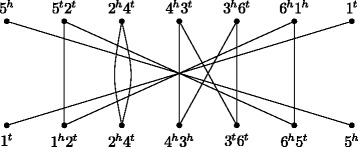
The adjacency graph for the genomes $A =\left \{\left (\circ \;{-5}\;2\;4\;3\;6\;{-1}\;\circ \right)\right \}$ and $B =\left \{\left (\circ \;1\;2\;4\;{-3}\;6\;5\;\circ \right)\right \}$

In most versions of the family-based setting the two genomes *A* and *B* have the same content, that is, $\mathcal {A} = \mathcal {B}$. When in addition there are no duplicates, that is, when there is exactly one representative of each family in each genome, we can easily build the *adjacency graph* of genomes *A* and *B*, denoted by *AG*(*A,B*) [6]. It is a bipartite multigraph such that each partition corresponds to the set of adjacencies of one of the two input genomes, and an edge connects the same extremities of genes in both genomes. In other words, there is a one-to-one correspondence between the set of edges in *AG*(*A,B*) and the set of gene extremities. Since the graph is bipartite and vertices have degree one or two, the adjacency graph is a collection of paths and even cycles. An example of an adjacency graph is presented in Fig. 1.

It is well known that a DCJ operation that modifies *AG*(*A,B*) by increasing either the number of even cycles by one or the number of odd paths by two decreases the DCJ distance between genomes *A* and *B* [6]. This type of DCJ operation is said to be *optimal*. Conversely, if we are interested in a DCJ similarity measure between *A* and *B*, rather than a distance measure, then it should be increased by such an optimal DCJ operation. This suggests that a formula for a DCJ similarity between two genomes should correlate to the number of connected components (in the following just *components*) of the corresponding adjacency graph.

When the genomes *A* and *B* are identical, their corresponding adjacency graph is a collection of *c* 2-cycles and *b* 1-paths [6], so that $c + \frac {b}{2} = |\protect \mathcal {A}|=|\protect \mathcal {B}|$. This should be the upper bound of our DCJ similarity measure, and the contribution of each component in the formula should be upper bounded by 1.

We know that an optimal operation can always be applied to adjacencies that belong to one of the two genomes and to one single component of *AG*(*A,B*), until the graph becomes a collection of 2-cycles and 1-paths. In other words, each component of the graph can be *sorted*, that is, converted into a collection of 2-cycles and 1-paths independently of the other components. Furthermore, it is known that each of the following components – an even cycle with 2*d*+2 edges, or an odd path with 2*d*+1 edges, or an even path with 2*d* edges – can be sorted with exactly *d* optimal DCJ operations. Therefore, for the same *d*, components with more edges should actually have higher contributions in the DCJ similarity formula.

With all these considerations, the contribution of each component *C* in the formula is then defined to be its *normalized length*$\widehat {\ell }(C)$:

$$\begin{array}{*{20}l} \widehat{\ell}(C) =\left\{ \begin{array}{ll} \frac{|C|}{|C|} = 1\:, & \text{if}\; C\; \text{is a cycle}, \\ \frac{|C|}{|C|+1}\:, & \text{if}\; C\; \text{is an odd path}, \\ \frac{|C|}{|C|+2}\:, & \text{if}\; C\; \text{is an even path}. \end{array}\right. \end{array} $$

Let be the set of all components in *AG*(*A,B*). The formula for the family-based DCJ similarity is the sum of their normalized lengths:

1 $$ \mathrm{s}_{\textup{\textsc{dcj}}}(A,B) = \sum_{C \in \cal{C}}\widehat{\ell}(C).  $$

Observe that s_DCJ_(*A,B*) is a positive value, indeed upper bounded by $|\protect \mathcal {A}|$ (or, equivalently, by $|\protect \mathcal {B}|$). In Fig. 1 the DCJ similarity is $\text {s}_{\text {\textsc {dcj}}}(A, B) = 2\cdot \frac {1}{2} + 3\cdot 1=4$. The formula of Eq. 1 is the family-based version of the family-free DCJ similarity defined in [17], as we will see in the following subsections.

### Gene similarity graph

In the family-free setting, each gene in each genome is represented by a unique (signed) symbol, thus $\protect \mathcal {A} \cap \protect \mathcal {B} = \emptyset $ and the cardinalities $|\mathcal {A}|$ and $|\mathcal {{B}}|$ may be distinct. Let *a* be a gene in *A* and *b* be a gene in *B*, then their *normalized gene similarity* is given by some value *σ*(*a,b*) such that 0≤*σ*(*a,b*)≤1.

**Fig. 2:**
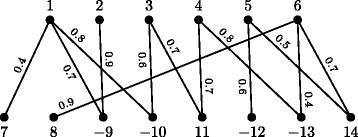
Representation of a gene similarity graph *GS*_*σ*_(*A,B*) for two unichromosomal linear genomes $A =\left \{\left (\circ \;1\;2\;3\;4\;5\;6\;\circ \right)\right \}$ and $B =\left \{\left (\circ \;7\;8\;{-9}\;{-10}\;11\;{-12}\;{-13}\;14\;\circ \right)\right \}$

We can represent the gene similarities between the genes of genome *A* and the genes of genome *B* with respect to *σ* in the so called *gene similarity graph* [15], denoted by *GS*_*σ*_(*A,B*). This is a weighted bipartite graph whose partitions $\protect \mathcal {A}$ and $\protect \mathcal {B}$ are the sets of (signed) genes in genomes *A* and *B*, respectively. Furthermore, for each pair of genes (*a,b*) such that $a \in \protect \mathcal {A}$ and $b \in \protect \mathcal {B}$, if *σ*(*a,b*)>0 then there is an edge *e* connecting *a* and *b* in *GS*_*σ*_(*A,B*) whose weight is *σ*(*e*):=*σ*(*a,b*). An example of a gene similarity graph is given in Fig. 2.

### Weighted adjacency graph

**Fig. 3:**
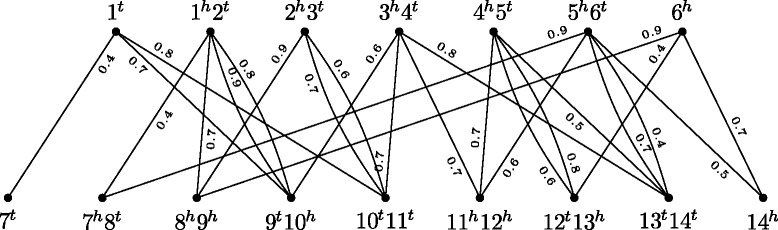
The weighted adjacency graph *AG*_*σ*_(*A,B*) for two unichromosomal linear genomes $A =\left \{\left (\circ \;1\;2\;3\;4\;5\;6\;\circ \right)\right \}$ and $B =\left \{\left (\circ \;7\;8\;{-9}\;{-10}\;11\;{-12}\;{-13}\;14\;\circ \right)\right \}$

The *weighted adjacency graph* *AG*_*σ*_(*A,B*) of two genomes *A* and *B* has a vertex for each adjacency in *A* and a vertex for each adjacency in *B*. For a gene *a* in *A* and a gene *b* in *B* with gene similarity *σ*(*a,b*)>0 there is one edge *e*^*h*^ connecting the vertices containing the two heads *a*^*h*^ and *b*^*h*^ and one edge *e*^*t*^ connecting the vertices containing the two tails *a*^*t*^ and *b*^*t*^. The weight of each of these edges is $\sigma \left (e^{h}\right) = \sigma \left (e^{t}\right) = \sigma (a,b)$. Differently from the simple adjacency graph, the weighted adjacency graph cannot be easily decomposed into cycles and paths, since its vertices can have degree greater than 2. As an example, the weighted adjacency graph corresponding to the gene similarity graph of Fig. 2 is given in Fig. 3.

We denote by *w*(*G*) the weight of a graph or subgraph *G*, that is given by the sum of the weights of all its edges, that is, $w(G) = \sum _{e \in G} \sigma (e)$. Observe that, for each edge *e*∈*GS*_*σ*_(*A,B*), we have two edges of weight *σ*(*e*) in *AG*_*σ*_(*A,B*), thus, the total weight of the weighted adjacency graph is $w\left (AG_{\sigma }(A,B)\right) = 2\,w\left (GS_{\sigma }(A,B)\right)$.

### Reduced genomes

Let *A* and *B* be two genomes and let *GS*_*σ*_(*A,B*) be their gene similarity graph. Now let *M*={*e*_1_,*e*_2_,…,*e*_*n*_} be a matching in *GS*_*σ*_(*A,B*) and denote by $w(M) = \sum _{e_{i} \in M} \sigma (e_{i})$ the weight of *M*, that is the sum of its edge weights. Since the endpoints of each edge *e*_*i*_=(*a,b*) in *M* are not saturated by any other edge of *M*, we can unambiguously define the function *ℓ*^*M*^(*a*)=*ℓ*^*M*^(*b*)=*i* to relabel each vertex in *A* and *B* [17]. The *reduced genome* *A*^*M*^ is obtained by deleting from *A* all genes not saturated by *M*, and renaming each saturated gene *a* to *ℓ*^*M*^(*a*), preserving its orientation (sign). Similarly, the reduced genome *B*^*M*^ is obtained by deleting from *B* all genes that are not saturated by *M*, and renaming each saturated gene *b* to *ℓ*^*M*^(*b*), preserving its orientation. Observe that the set of genes in *A*^*M*^ and in *B*^*M*^ is $\protect \mathcal {G}(M) = \left \{ \ell ^{M}(g) : g \text { is saturated by the matching} ~M \right \} = \{1,2,\ldots,n\}$.

### Weighted adjacency graph of reduced genomes

Let *A*^*M*^ and *B*^*M*^ be the reduced genomes for a given matching *M* of *GS*_*σ*_(*A,B*). The weighted adjacency graph $AG_{\sigma }\left (A^{M},B^{M}\right)$ can be obtained from *AG*_*σ*_(*A,B*) by deleting all edges that are not elements of *M* and relabeling the adjacencies according to *ℓ*^*M*^. Vertices that have no connections are then also deleted from the graph. Another way to obtain the same graph is building the adjacency graph of *A*^*M*^ and *B*^*M*^ and adding weights to the edges as follows. For each gene *i* in $\protect \mathcal {G}(M)$, both edges *i*^*t*^*i*^*t*^ and *i*^*h*^*i*^*h*^ inherit the weight of edge *e*_*i*_ in *M*, that is, $\sigma \left (i^{t}i^{t}\right) = \sigma \left (i^{h}i^{h}\right) = \sigma (e_{i})$. Consequently, the graph $AG_{\sigma }\left (A^{M},B^{M}\right)$ is also a collection of paths and even cycles and differs from $AG\left (A^{M}, B^{M}\right)$ only by the edge weights.

**Fig. 4:**
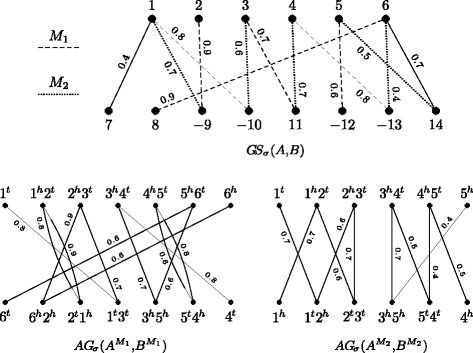
Considering, as in Fig. 2, the genomes $A =\left \{\left (\circ \;1\;2\;3\;4\;5\;6\;\circ \right)\right \}$ and $B =\left \{\left (\circ \;7\;8\;{-9}\;{-10}\;11\;{-12}\;{-13}\;14\;\circ \right)\right \}$, let *M*_1_ (dashed edges) and *M*_2_ (dotted edges) be two distinct maximal matchings in *GS*_*σ*_(*A,B*), shown in the upper part. The two resulting weighted adjacency graphs $AG_{\sigma }\left (A^{M_{1}},B^{M_{1}}\right)$, that has two cycles and two even paths, and $AG_{\sigma }\left (A^{M_{2}},B^{M_{2}}\right)$, that has two odd paths, are shown in the lower part

For each edge *e*∈*M*, we have two edges of weight *σ*(*e*) in $AG_{\sigma }\left (A^{M},B^{M}\right)$, therefore $w\left (AG_{\sigma }\left (A^{M}, B^{M}\right)\right) = 2\,w(M)$. Examples of weighted adjacency graphs of reduced genomes are shown in Fig. 4.

### The family-free DCJ similarity

For a given matching *M* in *GS*_*σ*_(*A,B*), a first formula for the weighted DCJ (wDCJ) similarity *s*_*σ*_ of the reduced genomes *A*^*M*^ and *B*^*M*^ was proposed in [15] only considering the cycles of $AG_{\sigma }\left (A^{M},B^{M}\right)$. After that, this definition was modified and extended in [17], in order to consider all components of the weighted adjacency graph.

First, let the *normalized weight*$\widehat {w}(C)$ of a component *C* of $AG_{\sigma }\left (A^{M},B^{M}\right)$ be:

$$\begin{array}{*{20}l} \widehat{w}(C) =\left\{ \begin{array}{ll} \frac{w(C)}{|C|}\:, & \text{if}\; C\; \text{is a cycle}\:, \\ \frac{w(C)}{|C|+1}\:, & \text{if}\; C\; \text{is an odd path}\:, \\ \frac{w(C)}{|C|+2}\:, & \text{if}\; C\; \text{is an even path}\:. \end{array}\right. \end{array} $$

Let be the set of all components in $AG_{\sigma }\left (A^{M},B^{M}\right)$. Then the wDCJ similarity *s*_*σ*_ is given by the following formula [17]:

2 $$ s_{\sigma}\left(A^{M},B^{M}\right) = \sum_{C \in \cal{C}}\widehat{w}(C)\:.  $$

Observe that, when the weights of all edges in *M* are equal to 1, this formula is equivalent to the one in Eq. 1.

The goal now is to compute the family-free DCJ similarity, i.e., to find a matching in *GS*_*σ*_(*A,B*) that maximizes *s*_*σ*_. However, although $s_{\sigma }\left (A^{M},B^{M}\right)$ is a positive value upper bounded by |*M*|, the behaviour of the wDCJ similarity does not correlate with the size of the matching, since smaller matchings, that possibly discard gene assignments, can lead to higher wDCJ similarities [17]. For this reason, the wDCJ similarity function is restricted to *maximal matchings* only, ensuring that no pair of genes with positive gene similarity score is simply discarded, even though it might decrease the overall wDCJ similarity. We then have the following optimization problem:

**Problem** FFDCJ-SIMILARITY(*A,B*): Given genomes *A* and *B* and their gene similarities *σ*, calculate their family-free DCJ similarity

3 $$ \textup{s}_{\textup{\textsc{ffdcj}}}(A, B) = \max_{M \in \mathbb{M}}\left\{ s_{\sigma}\left(A^{M},B^{M}\right) \right\},  $$

where $\mathbb {M}$ is the set of all maximal matchings in *GS*_*σ*_(*A,B*).

Problem FFDCJ-SIMILARITY is NP-hard [17]. Moreover, one can directly correlate the problem to the adjacency similarity problem, where the goal is to maximize the number of preserved adjacencies between two given genomes [11, 19]. However, since there the objective is to maximize the number of cycles of length 2, even an approximation for the adjacency similarity problem is not a good algorithm for the FFDCJ-SIMILARITY problem, where cycles of higher lengths are possible in the solution [20].

### Capping telomeres

A very useful preprocessing to *AG*_*σ*_(*A,B*) is the *capping* of telomeres, a general technique for simplifying algorithms that handle genomes with linear chromosomes, commonly used in the context of family-based settings [4, 5, 21]. Given two genomes *A* and *B* with *i* and *j* linear chromosomes, respectively, for each vertex representing only one extremity we add a *null extremity* *τ* to it (e.g., 1^*t*^ of Fig. 4 becomes *τ*1^*t*^). Furthermore, in order to add the same number of null extremities to both genomes, |*j*−*i*|*null adjacencies* *ττ* (composed of two null extremities) are added to genome *A*, if *i*<*j*, or to genome *B*, if *j*<*i*. Finally, for each null extremity of a vertex in *A* we add to *AG*_*σ*_(*A,B*) a *null edge* with weight 0 to each null extremity of vertices in *B*. Consequently, after capping of telomeres the graph *AG*_*σ*_(*A,B*) has no vertex of degree one. Notice that, if before the capping *p* was a path of weight *w* connecting telomeres in *AG*_*σ*_(*A,B*), then after the capping *p* will be part of a cycle closed by null extremities with normalized weight $\frac {w}{|p|+1}$ if *p* is an odd path, or of normalized weight $\frac {w}{|p|+2}$ if *p* is an even path. In any of the two cases, the normalized weight is consistent with the wDCJ similarity formula in Eq. 2.

## Results and discussion

### An exact Algorithm

In order to exactly compute the family-free DCJ similarity between two given genomes, we propose an integer linear program (ILP) formulation that is similar to the one for the family-free DCJ distance given in [17]. It adopts the same notation and also uses an approach to solve the maximum cycle decomposition problem as in [13].

Let *A* and *B* be two genomes, let *G*=*GS*_*σ*_(*A,B*) be their gene similarity graph, and let *X*_*A*_ and *X*_*B*_ be the extremity sets (including null extremities) with respect to *A* and *B* for the capped adjacency graph *AG*_*σ*_(*A,B*), respectively. The weight *w*(*e*) of an edge *e* in *G* is also denoted by *w*_*e*_. For the ILP formulation, an extension *H*=(*V*_*H*_,*E*_*H*_) of the capped weighted adjacency graph *AG*_*σ*_(*A,B*) is defined such that $V_{H} = X_{A} \cup X_{B}$, and $E_{H} = E_{m} \cup E_{a} \cup E_{s}$ has three types of edges: (*i*) *matching edges* that connect two extremities in different extremity sets, one in *X*_*A*_ and the other in *X*_*B*_, if they are null extremities or there exists an edge connecting these genes in *G*; the set of matching edges is denoted by *E*_*m*_; (*ii*) *adjacency edges* that connect two extremities in the same extremity set if they form an adjacency; the set of adjacency edges is denoted by *E*_*a*_; and (*iii*) *self edges* that connect two extremities of the same gene in an extremity set; the set of self edges is denoted by *E*_*s*_. Matching edges have weights defined by the normalized gene similarity *σ*, all adjacency and self edges have weight 0. Notice that any edge in *G* corresponds to two matching edges in *H*.

The description of the ILP follows. For each edge *e* in *H*, we create a binary variable *x*_*e*_ to indicate whether *e* will be in the final solution. We require first that each adjacency edge be chosen:

$$x_{e} = 1, \qquad \forall~e \in E_{a}. $$

Now we rename each vertex in *H* such that *V*_*H*_={*v*_1_,*v*_2_,…,*v*_*k*_} with *k*=|*V*_*H*_|. We require that each of these vertices be adjacent to exactly one matching or self edge:

$$\begin{aligned} \sum_{e = v_{r}v_{t} \in E_{m} \cup E_{s}} x_{e} = 1,& \forall~v_{r} \in X_{A}, \quad \text{and}\\ \sum_{e = v_{r}v_{t} \in E_{m} \cup E_{s}} x_{e} = 1,& \forall~v_{t} \in X_{B}. \end{aligned} $$

Then, we require that the final solution be valid, meaning that if one extremity of a gene in *A* is assigned to an extremity of a gene in *B*, then the other extremities of these two genes have to be assigned as well:

$$x_{a^{h}b^{h}} = x_{a^{t}b^{t}}, \qquad \forall~ab \in E_{G}. $$

We also require that the matching be maximal. This can easily be ensured if we guarantee that at least one of the vertices connected by an edge in the gene similarity graph be chosen, which is equivalent to not allowing both of the corresponding self edges in the weighted adjacency graph be chosen:

$$x_{a^{h}a^{t}} + x_{b^{h}b^{t}} \leq 1, \qquad \forall~ab \in E_{G}. $$

To count the number of cycles, we use the same strategy as described in [13]. For each vertex *v*_*i*_ we define a variable *y*_*i*_ that labels *v*_*i*_ such that

$$0 \leq y_{i} \leq i, \qquad 1 \leq i \leq k. $$

We also require that adjacent vertices have the same label, forcing all vertices in the same cycle to have the same label:

$$\begin{array}{*{20}l} y_{i} \leq y_{j} + i \cdot (1 - x_{e}), & \qquad \forall~e = v_{i}v_{j} \in E_{H}, \\ y_{j} \leq y_{i} + j \cdot (1 - x_{e}), &\qquad \forall~e = v_{i}v_{j} \in E_{H}. \end{array} $$

We create a binary variable *z*_*i*_, for each vertex *v*_*i*_, to verify whether *y*_*i*_ is equal to its upper bound *i*:

$$i\cdot z_{i} \leq y_{i}, \qquad 1 \leq i \leq k. $$

Since all variables *y*_*i*_ in the same cycle have the same label but a different upper bound, only one of the *y*_*i*_ can be equal to its upper bound *i*. This means that *z*_*i*_ is 1 if the cycle with vertex *i* as representative is used in a solution.

Now, let *L*={2*j*:*j*=1,…,*n*} be the set of possible cycle lengths in *H*, where $n := \min (|A|, |B|)$. We create the binary variable *x*_*ei*_ to indicate whether *e* is in *i*, for each *e*∈*E*_*H*_ and each cycle *i*. We also create the binary variable $x_{ei}^{\ell }$ to indicate whether *e* belongs to *i* and the length of cycle *i* is *ℓ*, for each *e*∈*E*_*H*_, each cycle *i*, and each *ℓ*∈*L*.

We require that if an edge *e* belongs to a cycle *i*, then it can be true for only one length *ℓ*∈*L*. Thus,

4 $$  \sum_{\ell \in L} x_{ei}^{\ell} \leq x_{ei}, \qquad \forall~e \in E_{H}\ \text{and}\ 1 \leq i \leq k.  $$

We create another binary variable $z_{i}^{\ell }$ to indicate whether cycle *i* has length *ℓ*. Then $\ell \cdot z_{i}^{\ell }$ is an upper bound for the total number of edges in cycle *i* of length *ℓ*:

$$\sum_{e \in E_{M}} x_{ei}^{\ell} \leq \ell \cdot z_{i}^{\ell}, \qquad \forall~\ell \in L\ \text{and}\ 1 \leq i \leq k. $$

The length of a cycle *i* is given by $\ell \cdot z_{i}^{\ell }$, for *i*=1,…,*k* and *ℓ*∈*L*. On the other hand, it is the total amount of matching edges *e* in cycle *i*. That is,

$$\sum_{\ell \in L} \ell \cdot z_{i}^{\ell} = \sum_{e \in E_{m}} x_{ei}, \qquad 1 \leq i \leq k. $$

We have to ensure that each cycle *i* must have just one length:

$$\sum_{\ell \in L} z_{i}^{\ell} = z_{i}, \qquad 1 \leq i \leq k. $$

Now we create the binary variable *y*_*ri*_ to indicate whether the vertex *v*_*r*_ is in cycle *i*. Thus, if *x*_*ei*_=1, i.e., if the edge *e*=*v*_*r*_*v*_*t*_ in *H* is chosen in cycle *i*, then *y*_*ri*_=1=*y*_*ti*_ (and *x*_*e*_=1 as well). Hence,

5 $$  \begin{aligned} \left. \begin{array}{rcl} x_{ei} & \leq & x_{e}, \\ x_{ei} & \leq & y_{ri}, \\ x_{ei} & \leq & y_{ti}, \\ x_{ei} & \geq & x_{e} + y_{ri} + y_{ti} - 2, \end{array} \right\} \quad \forall~e = v_{r}v_{t} \in E_{H} \text{ and}\ 1 \leq i \leq k. \end{aligned}  $$

Since *y*_*r*_ is an integer variable, we associate *y*_*r*_ to the corresponding binary variable *y*_*ri*_, for any vertex *v*_*r*_ belonging to cycle *i*:

$$y_{r} = \sum_{i = 1}^{r} i \cdot y_{ri}, \qquad \forall~v_{r} \in V_{H}. $$

Furthermore, we must ensure that each vertex *v*_*r*_ may belong to at most one cycle:

$$\sum_{i = 1}^{r} y_{ri} \leq 1, \qquad \forall~v_{r} \in V_{H}. $$

Finally, we set the objective function as follows:

$$\text{maximize} \quad \sum_{i = 1}^{k} \sum_{\ell \in L} \sum_{e \in E_{m}} \frac{w_{e}x_{ei}^{\ell}}{\ell}. $$

Note that, with this formulation, we do not have any path as a component. Therefore, the objective function above is exactly the family-free DCJ similarity S_FFDCJ_(*A,B*) as defined in Eqs. (2) and (3).

Notice that the ILP formulation has *O*(*N*^4^) variables and $O\left (N^{3}\right)$ constraints, where *N*=|*A*|+|*B*|. The number of variables is proportional to the number of variables $x_{ei}^{\ell }$, and the number of constraints is upper bounded by (4) and (5).

### APX-hardness and heuristics

In this section we first state that problem FFDCJ-SIMILARITY is APX-hard and provide a lower bound for the approximation ratio.

#### **Theorem 1**

FFDCJ-SIMILARITY is APX-hard and cannot be approximated with approximation ratio better than 22/21=1.0476…, unless P = NP.

#### *Proof*

See Additional file 1. □

We now propose four heuristic algorithms to compute the family-free DCJ similarity of two given genomes: one that is directly derived from a maximum matching of the gene similarity graph *GS*_*σ*_ and three greedy-like heuristics that, according to different criteria, select cycles from the weighted adjacency graph *AG*_*σ*_, such that the cycles selected by each heuristic induce a matching in *GS*_*σ*_.

#### Maximum matching

In the first heuristic, shown in Algorithm 1 (MAXIMUM-MATCHING), we find a maximum weighted bipartite matching *M* in *GS*_*σ*_ by the Hungarian Method, also known as Kuhn-Munkres Algorithm [22–24]. Given the matching *M*, it is straightforward to obtain the reduced genomes *A*^*M*^ and *B*^*M*^ and return the similarity value $s_{\sigma }\left (A^{M},B^{M}\right)$.



For the implementantion of this heuristic we cast similarity values (floating point edge weights in [0,1]) in *GS*_*σ*_(*A,B*) to integers by multiplying them by some power of ten, depending on the precision of similarity values. Given real or general simulated instances, and for a power of ten large enough, this operation has little impact on the optimality of the weighted matching *M* for the original weights in *GS*_*σ*_(*A,B*) obtained from the Kuhn-Munkres algorithm, i.e., the weight of *M* for the original weights in *GS*_*σ*_(*A,B*) is optimal or near-optimal since only less significant digits are not considered.

#### Greedy heuristics

Before describing the greedy heuristics, we need to introduce the following concepts. We say that two edges in *AG*_*σ*_(*A,B*) are *consistent* if one connects the head and the other connects the tail of the same pair of genes, or if they connect extremities of distinct genes in both genomes. Otherwise they are *inconsistent*. A set of edges, in particular a cycle, is consistent if it has no pair of inconsistent edges. A set of cycles is consistent if the union of all of their edges is consistent. Observe that a consistent set of cycles in *AG*_*σ*_(*A,B*) induces a matching in *GS*_*σ*_(*A,B*).

Each one of the three greedy algorithms selects disjoint and consistent cycles in the capped *AG*_*σ*_(*A,B*). The consistent cycles are selected from the set of all cycles of *AG*_*σ*_(*A,B*), that is obtained in Step 4 of each heuristic (see Algorithms 2, 3 and 4 below), using a cycle enumeration algorithm by Hawick and James [25], which is based on Johnson’s algorithm [26]. For this reason, the running time of our heuristics is potentially exponential in the number of vertices of *AG*_*σ*_(*A,B*).

In the three heuristics, after completing the cycle selection by iterating over the set of all cycles of *AG*_*σ*_(*A,B*), the induced matching *M* in *GS*_*σ*_(*A,B*) could still be non-maximal. Whenever this occurs, among the genes that are unsaturated by *M*, we can identify *disposable genes* by one of the two following conditions:

1. Any unsaturated gene in *GS*_*σ*_(*A,B*) that is connected only to saturated genes, is a disposable gene;
2. For a given set of vertices $S \subseteq \protect \mathcal {A}$ (or $S \subseteq \protect \mathcal {B}$) in *GS*_*σ*_(*A,B*) such that, for the set of connected genes N(*S*), we have |*S*|>|N(*S*)| (Hall’s theorem), then any subset of size |*S*|−|N(*S*)| of unsaturated genes of *S* can be set as disposable genes. In our implementation we choose those |*S*|−|N(*S*)| unsaturated genes with the smallest labels. Such $S \subseteq \protect \mathcal {A}$ can be found as follows. Let *v* be the set of vertices saturated by *M*, and let *M*^′^ be a maximum cardinality matching in *GS*_*σ*_(*A,B*)∖*v*. Consider the sets $\protect \mathcal {A}' = \protect \mathcal {A} \setminus v$ and $\protect \mathcal {B}' = \protect \mathcal {B} \setminus v$. Now let *GSσ*′(*A,B*) be a directed bipartite graph on the vertex set $\protect \mathcal {A}' \cup \protect \mathcal {B}'$, which includes the edges of *M*^′^ oriented from $\protect \mathcal {B}'$ to $\protect \mathcal {A}'$ and the remaining edges of *GS*_*σ*_(*A,B*)∖*v* oriented from $\protect \mathcal {A}'$ to $\protect \mathcal {B}'$, and let $U \subseteq \protect \mathcal {A}'$ be the set of vertices of $\protect \mathcal {A}'$ unsaturated by *M*^′^. $S \subseteq \protect \mathcal {A}$ is the corresponding set of vertices reachable from *U* in *GSσ*′(*A,B*), if any. $S \subseteq \protect \mathcal {B}$ can be found analogously.

**Fig. 5:**
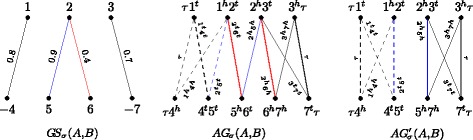
Consider genomes $A =\left \{\left (\circ \;1\;2\;3\;\circ \right)\right \}$ and $B =\left \{\left (\circ \;{-4}\;5\;6\;{-7}\;\circ \right)\right \}$ and their gene similarity graph *GS*_*σ*_(*A,B*). The selection of the dashed cycle in *AG*_*σ*_(*A,B*) adds to the matching *M* in *GS*_*σ*_(*A,B*) the edges connecting gene 1 to gene 4 and gene 2 to gene 5. After this selection, although the matching *M* is not yet maximal, there are no more consistent cycles in *AG*_*σ*_(*A,B*). Observe that in *GS*_*σ*_(*A,B*) gene 6 is unsaturated and its single neighbor - gene 2 - is already saturated. Since gene 6 can no longer be saturated by *M*, it is a disposable gene and is deleted from *AG*_*σ*_(*A,B*), resulting in *AGσ*′(*A,B*), where a new consistent cycle appears. The selection of this new cycle adds to the matching *M* the edge connecting gene 3 to gene 7. Both *AG*_*σ*_(*A,B*) and *AGσ*′(*A,B*) have a simplified representation, in which the edge weights, as well as two of the four null edges of the capping, are omitted. Furthermore, for the sake of clarity, in this simplified representation each edge has a label describing the extremities connected by it

If there is no consistent cycle to be selected and the matching *M* is still non-maximal, new consistent cycles appear in *AG*_*σ*_(*A,B*) after the deletion of all identified disposable genes (see Fig. 5). In order to delete a disposable gene *g*, we need to remove from *AG*_*σ*_(*A,B*) the edges corresponding to extremities *g*^*t*^ or *g*^*h*^ and “merge” the two vertices that represent these extremities. Every time disposable genes are deleted from *AG*_*σ*_(*A,B*), a new iteration of the algorithms starts from Step 4 (see again Algorithms 2, 3 and 4). This procedure assures that, in each one of the three algorithms, the final set of selected cycles defines a maximal matching *M*, such that $AG_{\sigma }\left (A^{M},B^{M}\right)$ is exactly the union of those selected cycles.

**Best density**

The best density heuristic is shown in Algorithm 2 (GREEDY-DENSITY). The *density* of a cycle *C* is given by $\frac {w(C)}{|C|^{2}}$ (its weight divided by the square of its length). The cycles of *AG*_*σ*_(*A,B*) are arranged in decreasing order of their densities, and consistent cycles are selected following this order.



Since the number of cycles of any length may be exponential in the size of the input graph, in our implementation we add a heuristic in which initially the search is restricted to cycles of length up to ten. Then, as long as the obtained matching is not maximal, Steps 4 to 7 are repeated, while gradually increasing the allowed maximum cycle length in steps of ten.

**Best length**

The best length heuristic is shown in Algorithm 3 (GREEDY-LENGTH). The cycles of *AG*_*σ*_(*A,B*) are found in increasing order of their lengths, and ties are broken by the decreasing order of their weights. Here we first find and select cycles of length 2, then of length 4, and so on, for each fixed length iterating over the set of all cycles in decreasing order of their weights. Consistent cycles are selected following this procedure.



**Best length with weighted maximum independent set**

The best length heuristic with WMIS is shown in Algorithm 4 (GREEDY-WMIS) and is a variation of GREEDY-LENGTH. Instead of selecting cycles of greater weights for a fixed length, this algorithm selects the greatest amount of cycles for a fixed length by a WMIS algorithm. The heuristic builds a *cycle graph* where each vertex is a cycle of *AG*_*σ*_(*A,B*), the weight of a vertex is the weight of the cycle it represents and two vertices are adjacent if the cycles they represent are inconsistent. The heuristic tries to find next an independent set with the greatest weight in the cycle graph. Since this graph is not *d*-claw-free for any fixed *d*, the WMIS algorithm [27] does not guarantee any fixed ratio.



### Experimental results

Experiments for the ILP and our heuristics were conducted on an Intel i7-4770 3.40GHz machine with 16 GB of memory. In order to do so, we produced simulated datasets by the Artificial Life Simulator (ALF) [28] and obtained real genome data from NCBI, using the FFGC tool [29] to obtain similarity scores between genomes. Gurobi Optimizer 7.0 was set to solve ILP instances with default parameters, time limit of 1800 s and 4 threads, and the heuristics were implemented in C++.

#### Simulated data

We generated datasets with 10 genome samples each, running pairwise comparisons between all genomes in the same dataset. Each dataset has genomes of sizes around 25, 50 or 1000 (the latter used only for running the heuristics), generated based on a sample from the tree of life with 10 leaf species and PAM distance of 100 from the root to the deepest leaf. Gamma distribution with parameters *k*=3 and *θ*=133 was used for gene length distribution. For amino acid evolution we used the WAG substitution model with default parameters and the preset of Zipfian indels with rate 0.00005. Regarding genome level events, we allowed gene duplications and gene losses with rate 0.002, and reversals and transpositions (which ALF refers to as translocations) with rate 0.0025, with at most 3 genes involved in each event. To test different proportions of genome level events, we also generated simulated datasets with 2- and 5-fold increase for reversal and transpositions rates.

Results are summarized in Table 1. Each dataset is composed of 10 genomes, totaling 45 comparisons of pairs per dataset. Rate *r*=1 means the default parameter set for genome level events, while *r*=2 and *r*=5 mean the 2- and 5-fold increase of rates, respectively. For the ILP the table shows the average time for instances for which an optimal solution was found, the number of instances for which the optimizer did not find an optimal solution within the given time limit and, for the latter class of instances, the average relative gap between the best solution found and the upper bound found by the solver, calculated by $\left (\frac {\text {upper bound}}{\text {best solution}} - 1\right) \times 100$. For our heuristics, the running time for all instances of sizes 25 and 50 was negligible, therefore the table shows only the average relative gap between the solution found and the upper bound given by the ILP solver (if any).

**Table 1:** Results of experiments for simulated genomes

	ILP			Maximum-Matching	Greedy-Density	Greedy-Length	Greedy-wmis
	Time (s)	Not finished	Gap (%)	Gap (%)	Gap (%)	Gap (%)	Gap (%)
25 genes, *r*=1	19.50	0	–	16.26	5.03	5.84	5.97
25 genes, *r*=2	84.60	2	69.21	58.69	30.77	43.57	43.00
25 genes, *r*=5	49.72	0	–	81.39	43.83	55.38	55.38
50 genes, *r*=1	265.23	12	23.26	63.02	24.76	27.86	26.94
50 genes, *r*=2	463.50	29	38.12	123.71	65.41	66.52	64.78
50 genes, *r*=5	330.88	29	259.72	281.70	177.58	206.60	206.31

Results clearly show the average relative gap of heuristics increases proportionally to the rate of reversals and transpositions. This is expected, as higher mutation rates often result in higher normalized weights on longer cycles, thus the association of genes with greater gene similarity scores will be subject to the selection of longer cycles. Interestingly, for some larger instances the relative gap for heuristics is very close to the values obtained by the ILP solver, suggesting the use of heuristics may be a good alternative for some classes of instances or could help the solver finding lower bounds quickly. It is worth noting that the GREEDY-DENSITY heuristic found solutions with gap smaller than 1% for 38% of the instances with 25 genes.

In a single instance (25 genes, *r*=2), the gap between the best solution found and the upper bound was much higher for the ILP solver and for the heuristics. This instance in particular is precisely the one with the largest number of edges in *GS*_*σ*_(*A,B*) in the dataset. This may indicate that a moderate increase in degree of vertices (1.3 on average to 1.8 in this case) may result in much harder instances for the solver and the heuristics, as after half of the time limit the solver attained no significant improvement on solutions found, and the heuristics returned solutions with a gap even higher.

We also simulated 10 genomes of sizes around 50, with PAM distance of 15 from the root to the deepest leaf, therefore evolutionarily “closer” to each other and for which higher similarity values are expected. For these genomes the default rates were multiplied by ten (10-fold) for Zipfian indels, gene duplications, gene losses, reversals and transpositions, otherwise there would be no significative difference between them. The exact ILP algorithm found an optimal solution for only 4 of the 45 instances, taking 840.59 s on average. For the remaining instances, where the ILP did not finish within the time limit, the average gap is 329.53%. Regarding the heuristics (Table 2), that all run in negligible time, GREEDY-DENSITY outperforms the others, with an average gap of 163% compared to the best upper bound found by the ILP solver. Surprisingly, values returned by greedy heuristics are better than values obtained by the ILP for these instances. Results again suggest that the ILP could benefit greatly from heuristics by using their results as initial lower bounds. Moreover, for some groups of instances even heuristics alone can obtain excellent results.

**Table 2:** Results of experiments for 10 simulated genomes (45 pairwise comparisons) with smaller PAM distance

	ILP			Maximum-Matching	Greedy-Density	Greedy-Length	Greedy-wmis
	Time (s)	Not finished	Gap (%)	Gap (%)	Gap (%)	Gap (%)	Gap (%)
50 genes, *r*=10	840.59	41	329.53	415.57	163.00	172.02	168.58

Although we have no upper bounds for comparing the results of our heuristics for genome sizes around 1000, they are still very fast. For these genomes we analyze the MAXIMUM-MATCHING algorithm separately afterwards, taking into account for now only the other three heuristics. The average running times are 0.30 s, 15.11 s and 12.16 s for GREEDY-DENSITY, GREEDY-LENGTH and GREEDY-WMIS, respectively, showing nevertheless little difference on results.

However, in 25% of the instances with *r*=5, the difference from the best to the worst solutions provided by these heuristics varied between 10% and 24%, the best of which were given by GREEDY-DENSITY. That is probably because, instead of prioritizing shorter cycles, GREEDY-DENSITY attempts to balance both normalized weight and length of the selected cycles. The average running times for the instances with *r*=5 are 1.84 s, 76.02 s and 80.67 s for GREEDY-DENSITY, GREEDY-LENGTH and GREEDY-WMIS, respectively.

Still for genomes of size around 1000 and *r*=5, the MAXIMUM-MATCHING heuristic is the fastest, with an average running time of 1.70 s. Despite being the best heuristic for a few cases, the similarity value given by this heuristic is merely 27% of the value given by the best heuristic, on average. While the MAXIMUM-MATCHING heuristic is clearly not useful for calculating similarity values, these results show how significant it is choose cycles with the best normalized weights versus prioritizing edges with best weights in the gene similarity graph for the FFDCJ-SIMILARITY problem. Since this property of the MAXIMUM-MATCHING somehow reflects the strategy of family-based comparative genomics, this observation indicates an advantage of family-free analysis compared to family-based analysis.

To better understand how cycles scale, we generated 5-fold larger instances (up to 10000 genes), running the GREEDY-DENSITY heuristic. Results show that most of the cycles found are of short lengths compared to the genome sizes and in practice their number does not increase exponentially, providing some insight on why our heuristics are fast.

Finally, as expected, experiments for genomes simulated with different parameters indicate the FFDCJ similarity decreases as the PAM distance or the rates of genome level events increases (data not shown).

#### Real genome data

To show the applicability of our methods to real data, we obtained from NCBI protein-coding genes of X chromosomes of human (*Homo-sapiens*, assembly GRCh38.p7), house mouse (*Mus musculus*, assembly GRCm38.p4 C57BL/6J), and Norway rat (*Rattus norvegicus*, assembly Rnor_6.0). In mammals, the set of genes on the X chromosome has been reasonably conserved throughout the last several million years [30], having however their order disrupted many times.

Since protein sequences are used to obtain the similarity scores (with the help of the BLASTp tool) instead of nucleotide sequences, 76 genes from the rat genome were excluded because no protein sequence was available. Besides, when a gene has multiple isoforms, the longest is kept. The number of genes in the resulting genomes were 822, 953 and 863 for human, mouse and rat, respectively, some of them removed from the pairwise genome comparison due to the pruning process of FFGC.

Table 3 shows, as expected, that the two rodent X chromosomes have a higher similarity than any of them to the human X chromosome. The values returned by the greedy heuristics are very similar, where GREEDY-LENGTH is the fastest. MAXIMUM-MATCHING results are less than 5% distant from the results of the greedy heuristics, which indicates the choice of cycles has some influence but does not dominate the similarity values obtained for these instances. Matching sizes are similar for all heuristics, showing that about 8% of the genes of the smaller genomes could not be matched to some gene of the other genome and had to be removed, that is, they are disposable genes.

**Table 3:** Results for heuristics on real genomes

	Smaller genome	Matching size				Time (s)				Similarity			
		MM	GD	GL	GW	MM	GD	GL	GW	MM	GD	GL	GW
Human/mouse	696	643	643	643	643	0.07	19.6	0.1	8.6	404.56	420.64	421.48	420.72
Human/rat	672	613	611	611	612	0.05	11.6	0.04	3.3	358.36	374.17	374.27	373.82
Mouse/rat	746	690	689	689	689	0.17	0.18	0.13	0.18	481.53	500.59	500.57	500.36

*Smaller genome* column shows for each pair of genomes the number of genes in the smaller one, an upper bound for the matching size. Heuristics are represented by their initials (e.g. Greedy-Length = GL)

## Conclusions

In this paper we developed methods for computing the (NP-hard) family-free DCJ similarity, which is a large-scale rearrangement measure for comparing two given genomes. We presented an exact algorithm in form of an integer linear program and extended our previous hardness result by showing that the problem is APX-hard and has a lower bound of 22/21 for its approximation ratio. Therefore, we developed four heuristic algorithms and could show that they perform well while having reasonable running times also for realistic-size genomes.

Our initial experiment on real data can be considered a proof of concept. In general, the computational results of this paper can be used to more systematically study the applicability of the DCJ similarity measure in various contexts. One important point to be investigated is whether, differently from parsimonious distance measures that usually only hold for closely related genomes, a genomic similarity would allow to perform good comparisons of more distant genomes as well. Fine-tuning of both the data preparation and objective function may be necessary, though.

For example, one drawback of the function s_FFDCJ_ as defined in Eq. 3 is that distinct pairs of genomes might give family-free DCJ similarity values that cannot be compared easily, because the value of S_FFDCJ_ varies between 0 and |*M*|, where *M* is the matching giving rise to S_FFDCJ_. Therefore some kind of normalization would be desirable. A simple approach could be to divide S_FFDCJ_ by the size of the smaller genome, because this is a trivial upper bound for |*M*|. Moreover, it can be applied as a simple postprocessing step, keeping all theoretical results of this paper valid. A better normalization, however, might be to divide by |*M*| itself. An analytical treatment here seems more difficult, though. Therefore we leave this and the application to multiple genomes in a phylogenetic context as an open problem for future work.

Other questions that can be studied in the future are the relationships between family-based and family-free genomic similarity measures in general.

## Additional file

Additional file 1 APX-hardness proof of the ffdcj-similarity problem. (PDF 379 kb)

## References

[1] Sankoff D. Edit distance for genome comparison based on non-local operations. In: Proc. of CPM 1992 LNCS, vol. 644.1992. p. 121–35.

[2] Sankoff D (1999). Genome rearrangement with gene families. Bioinformatics.

[3] Bafna V, Pevzner P (1993). Genome rearrangements and sorting by reversals. Proc. of FOCS 1993.

[4] Hannenhalli S, Pevzner P (1995). Transforming men into mice (polynomial algorithm for genomic distance problem). Proc. of FOCS 1995.

[5] Yancopoulos S, Attie O, Friedberg R (2005). Efficient sorting of genomic permutations by translocation, inversion and block interchanges. Bioinformatics.

[6] Bergeron A, Mixtacki J, Stoye J, Bucher P, Moret BME (2006). A unifying view of genome rearrangements. Proc. of WABI 2006. LNBI, vol. 4175.

[7] Braga MDV, Willing E, Stoye J (2011). Double cut and join with insertions and deletions. J Comput Biol.

[8] Bryant D, Sankoff D, Nadeau JH (2000). The complexity of calculating exemplar distances. Comparative Genomics.

[9] Bulteau L, Jiang M (2013). Inapproximability of (1,2)-exemplar distance. IEEE/ACM Trans. Comput. Biol. Bioinf.

[10] Angibaud S, Fertin G, Rusu I, Vialette S (2007). A pseudo-boolean framework for computing rearrangement distances between genomes with duplicates. J Comput Biol.

[11] Angibaud S, Fertin G, Rusu I, Thévenin A, Vialette S (2008). Efficient tools for computing the number of breakpoints and the number of adjacencies between two genomes with duplicate genes. J Comput Biol.

[12] Angibaud S, Fertin G, Rusu I, Thévenin A, Vialette S (2009). On the approximability of comparing genomes with duplicates. Journal of Graph Algorithms and Applications.

[13] Shao M, Lin Y, Moret B (2014). An exact algorithm to compute the DCJ distance for genomes with duplicate genes. Proc. of RECOMB 2014. LNBI.

[14] Doerr D, Thévenin A, Stoye J (2012). Gene family assignment-free comparative genomics. BMC Bioinformatics.

[15] Braga MDV, Chauve C, Doerr D, Jahn K, Stoye J, Thévenin A, Wittler R, Chauve C, El-Mabrouk N, Tannier E (2013). The potential of family-free genome comparison. Models and Algorithms for Genome Evolution.

[16] Durrett R, Nielsen R, York TL (2004). Bayesian estimation of genomic distance. Genetics.

[17] Martinez FV, Feijão P, Braga MDV, Stoye J (2015). On the family-free DCJ distance and similarity. Algoritm Mol Biol.

[18] Rubert DP, Medeiros GL, Hoshino EA, Braga MDV, Stoye J, Martinez FV (2017). Algorithms for computing the family-free genomic similarity under DCJ. Proc. of RECOMB-CG 2017. LNBI.

[19] Chen Z, Fu B, Xu J, Yang B, Zhao Z, Zhu B (2007). Non-breaking similarity of genomes with gene repetitions. Proc. of Combinatorial Pattern Matching (CPM 2007).

[20] Rubert DP, Feijão P, Braga MDV, Stoye J, Martinez FV (2017). Approximating the DCJ distance of balanced genomes in linear time. Algoritm Mol Biol.

[21] Shao M, Lin Y (2012). Approximating the edit distance for genomes with duplicate genes under DCJ, insertion and deletion. BMC Bioinformatics.

[22] Munkres J (1957). Algorithms for the assignment and transportation problems. J SIAM.

[23] Edmonds J, Karp RM (1972). Theoretical improvements in algorithmic efficiency for network flow problems. J ACM.

[24] Tomizawa N (1971). On some techniques useful for solution of transportation network problems. Networks.

[25] Hawick KA, James HA. Enumerating circuits and loops in graphs with self-arcs and multiple-arcs, Technical Report CSTN-013: Massey University; 2008.

[26] Johnson D (1975). Finding all the elementary circuits of a directed graph. SIAM J Comput.

[27] Berman P, Halldórsson MM (2000). A *d*/2 approximation for maximum weight independent set in *d*-claw free graphs. Proc. of SWAT 2000.

[28] Dalquen DA, Anisimova M, Gonnet GH, Dessimoz C (2012). Alf – a simulation framework for genome evolution. Mol Biol Evol.

[29] Doerr D. Family Free Genome Comparison (FFGC). 2017. https://bibiserv2.cebitec.uni-bielefeld.de/ffgc. Accessed 31 Jan 2018.

[30] Ohno S (2013). Sex Chromosomes and Sex-linked Genes. Endocrinology, vol. 1.

