# Supplementary material for: Computing the family-free DCJ similarity
Source: BMC Bioinformatics. 2018 May 8;19(Suppl 6):152. doi: 10.1186/s12859-018-2130-5 (PMC5998916; doi:10.1186/s12859-018-2130-5)
Supplement: Supplementary file 1 — APX-hardness proof of the ffdcj-similarity problem. (PDF 379 kb) [file 12859_2018_2130_MOESM1_ESM.pdf]

Computing the Family-Free DCJ Similarity

Diego P Rubert, Edna A Hoshino, Marília DV Braga, Jens Stoye and Fábio V Martinez

Additional file 1

This additional file contains the APX-hardness proof of problem FFDCJ-SIMILARITY. We first give some definitions based on [1]. Thereby we restrict ourselves to maximization problems and feasible solutions.

Given an instance x of an optimization problem P and a solution y of x, $\mathrm{val}(x, y)$ denotes the value of y, which is a positive integer measure of y. The function val, also referred to as objective function, must be computable in polynomial time. The value of an optimal solution (which maximizes the objective function) is defined as $\mathrm{opt}(x)$. Thus, the *performance ratio* of y with respect to x is defined as:

$$R_P(x, y) = \frac{\mathrm{opt}(x)}{\mathrm{val}(x, y)}. \tag{1}$$

Given two optimization problems P and P', let f be a polynomial-time computable function that maps an instance x of P into an instance $f(x)$ of P', and let g be a polynomial-time computable function that maps a solution y for the instance $f(x)$ of P' into a solution $g(x, y)$ of P. A *reduction* is a pair (f, g). A reduction from P to P' is frequently denoted by $P \leq P'$, and we say that P is *reduced* to P'. A reduction $P \leq P'$ *preserves membership* in a class $\mathcal{C}$ if $P' \in \mathcal{C}$ implies $P \in \mathcal{C}$. An *approximation-preserving* reduction preserves membership in either APX, PTAS, or both classes. The *strict reduction*, which is the simplest type of approximation-preserving reduction, preserves membership in both APX and PTAS classes and must satisfy the following condition:

$$R_P(x, g(x, y)) \leq R_{P'}(f(x), y). \tag{2}$$

We consider the following optimization problem, to be used within the proof of Theorem 1 below:

> **Problem** MAX-2SAT3(ϕ): Given a 2-CNF formula (i.e., with at most 2 literals per clause) $\phi = \{C_1, \ldots, C_m\}$ with n variables $X = \{x_1, \ldots, x_n\}$, where each variable appears in at most 3 clauses, find an assignment that satisfies the largest number of clauses.

The formula ϕ as defined above is called a 2SAT3 formula. MAX-2SAT3 [2, 3] is a special case of MAX-2SATB (also known as B-OCC-MAX-2SAT), where each variable occurs in at most B clauses for some B, which in turn is a restricted version of MAX-2SAT [4].

Theorem 1 FFDCJ-SIMILARITY *is APX-hard and cannot be approximated with approximation ratio better than* $22/21 = 1.0476\ldots$, *unless* $P = NP$.

Proof. [Theorem 1, first part] We give a strict reduction (f, g) from MAX-2SAT3 to FFDCJ-SIMILARITY, showing that

$$R_{\text{MAX-2SAT3}}(\phi, g(f(\phi), \gamma)) \leq R_{\text{FFDCJ-SIMILARITY}}(f(\phi), \gamma),$$

for any instance ϕ of MAX-2SAT3 and solution γ of FFDCJ-SIMILARITY with instance $f(\phi)$. Since variables occurring only once imply their clauses and others to be trivially satisfied, we consider only clauses that are not trivially satisfied in their instance. Similar for clauses containing literals x_i and $\overline{x_i}$, for some variable x_i.

(Function f.) We show progressively how to build $GS_\sigma(A,B)$ and define genes and their sequences in chromosomes of A and B. For each variable x_i occurring three times, let Cx_i^1, Cx_i^2 and Cx_i^3 be *aliases* for the clauses where x_i occurs (notice that a clause composed of two literals has two aliases). We define a *variable component* $\mathcal{C}_i$ adding vertices (genes) x_i^1, x_i^2 and x_i^3 to $\mathcal{A}$, vertices (genes) Cx_i^1, Cx_i^2 and Cx_i^3 to $\mathcal{B}$, and edges $ex_i^j = (Cx_i^j, x_i^j)$ and $e\overline{x_i}^j = (Cx_i^j, x_i^k)$ for $j \in \{1,2,3\}$ and $k = (j+1) \bmod 3 + 1$. An edge ex_i^j ($e\overline{x_i}^j$) has weight 1 if the literal x_i ($\overline{x_i}$) belongs to the clause Cx_i^j, otherwise it has weight 0. Edges in the variable component $\mathcal{C}_i$ form a cycle of length 6 (Fig. 1). Variable components for variables occurring two times are defined in a similar manner. Genomes are $A = \{(x_i^j)$ for each occurrence j of each variable $x_i \in X\}$ and $B = \{(Cx_i^j) : Cx_i^j$ is an alias to a clause in ϕ with only one literal$\} \cup \{(Cx_i^j\ Cx_{i'}^{j'}) : Cx_i^j$ and $Cx_{i'}^{j'}$ are aliases to the same clause in $\phi\}$.

The function f as defined here maps an instance ϕ of MAX-2SAT3 (a 2-CNF formula) to an instance $f(\phi)$ of FFDCJ-SIMILARITY (genomes A and B and $GS_\sigma(A,B)$) and is clearly polynomial. Besides, since all chromosomes are circular, the corresponding weighted adjacency graph $AG_\sigma(A,B)$ (or $AG_\sigma(A^M,B^M)$ for some matching M) is a collection of cycles only.

Now, notice that any maximal matching in $GS_\sigma(A,B)$ covers all genes in both $\mathcal{A}$ and $\mathcal{B}$, inducing in $AG_\sigma(A,B)$ only cycles of length 2, composed by (genes in) chromosomes (x_i^j) and $(Cx_i^{j'})$, or cycles of length 4, composed by chromosomes (x_i^j), (x_k^l) and $(Cx_i^{j'}\ Cx_k^{l'})$.

Recall that the normalized weight for a cycle C is $\widehat{w}(C) = \frac{w(C)}{|C|}$. In this transformation, each cycle C is such that $\widehat{w}(C) = 0, 0.5$ or 1. A cycle C such that $\widehat{w}(C) > 0$ is a *helpful cycle* and represents a clause satisfied by one or two literals ($\widehat{w}(C) = 0.5$ or $\widehat{w}(C) = 1$, respectively). See an example in Fig. 2.

In this scenario, however, a solution of FFDCJ-SIMILARITY with performance ratio r could lead to a solution of MAX-2SAT3 with ratio $2r$, since the total normalized weight for two cycles C_1 and C_2 with $\widehat{w}(C_1) = \widehat{w}(C_2) = 0.5$ (two clauses satisfied by one literal each) is the same for one cycle C with $\widehat{w}(C) = 1.0$ (one clause satisfied by two literals). Therefore, achieving the desired ratio requires some modifications in f. It is not possible to make these two types of cycles have the same weight, but it suffices to get close enough.

We introduce special genes into the genomes called *extenders*. For some p even, for each edge $ex_i^j = (Cx_i^j, x_i^j)$ of weight 1 in $GS_\sigma(A,B)$ we introduce p extenders $\alpha_1, \ldots, \alpha_p$ into A (as a consequence, they are also introduced into $\mathcal{A}$) and p extenders $\alpha_{p+1}, \ldots, \alpha_{2p}$ into B (each ex_i^j of weight 1 has its own set of extenders). Edge ex_i^j is replaced by edges (Cx_i^j, α_1) with weight 1 (which we consider equivalent to ex_i^j) and (α_{p+1}, x_i^j) with weight 0, and edges (α_k, α_{p+k}) with weight 0 are added to $GS_\sigma(A,B)$ for each $1 \leq k \leq p$ (extenders α_1 and α_{p+1} are now part of the variable component $\mathcal{C}_i$). Regarding new chromosomes in genomes A and B, A is updated to $A \cup \{(\alpha_1\ {-\alpha_p})\} \cup \{(\alpha_k\ {-\alpha_{k+1}}) : k \in \{2,4,\ldots,p-2\}\}$ and B to $B \cup \{(\alpha_k\ {-\alpha_{k+1}}) : k \in \{p+1, p+3, \ldots, 2p-1\}\}$. By this construction, which is

still polynomial, the path from x_i^{jt} to Cx_i^{jt} in $AG_\sigma(A,B)$ is extended from 1 to $1+p$ edges, from $\{(x_i^{jt}, Cx_i^{jt})\}$ to $\{(x_i^{jt}, \alpha_p^t), (\alpha_{p+1}^t, \alpha_2^t), (\alpha_3^t, \alpha_{p+2}^t), (\alpha_{p+3}^t, \alpha_4^t), \ldots, (\alpha_1^t, Cx_i^{jt})\}$. The same occurs for the path from x_i^{jh} to Cx_i^{jh} (see Fig. 3). Now, cycles in $AG_\sigma(A,B)$ induced by edges of weight 0 in $GS_\sigma(A,B)$ have normalized weight 0, cycles previously with normalized weight 1 are extended and have normalized weight $\frac{1}{1+p}$, and cycles previously with normalized weight 0.5 are extended and have normalized weight $\frac{1}{2+p}$. Notice that, for a sufficiently large p, $\frac{1}{1+p}$ is quite close to $\frac{1}{2+p}$, hence the problem of finding the maximum similarity in this graph is very similar to finding the maximum number of helpful cycles.

(Function g.) By the structure of variable components in $GS_\sigma(A,B)$, and since solutions of FFDCJ-SIMILARITY are restricted to maximal matchings only, any solution γ for $f(\phi)$ is a matching that covers only edges ex_i^j or $e\overline{x_i}^j$ for each variable component $\mathcal{C}_i$. For a $\mathcal{C}_i$, if edges ex_i^j ($e\overline{x_i}^j$) are in the solution then the variable x_i is assigned to true (false), inducing in polynomial time an assignment for each $x_i \in X$ and therefore a solution $g(f(\phi),\gamma)$ to MAX-2SAT3. A clause is satisfied if vertices (or the only vertex) corresponding to its aliases are in a helpful cycle.

(Approximation ratio.) Given $f(\phi)$ and a feasible solution γ of FFDCJ-SIMILARITY with the maximum number of helpful cycles, denote by c' the number of helpful cycles in γ. Notice that c' is also the maximum number of satisfied clauses of MAX-2SAT3, that is, the value of an optimal solution for MAX-2SAT3 for any instance ϕ, denoted here by $\text{opt}_{\text{2SAT3}}(\phi)$. Thus, $c' = \text{opt}_{\text{2SAT3}}(\phi)$.

To achieve the desired ratio we must establish some properties and relations between the parameters of MAX-2SAT3 and FFDCJ-SIMILARITY and set some parameters to specific values.

Let $n := |A| = |B|$ before extenders are added. We choose for p (the number of extenders added for each edge of weight 1 in $GS_\sigma(A,B)$) the value $2n$ and define $\omega = \frac{1}{2+p} = \frac{1}{2+2n}$ and

$$\varepsilon = \frac{1}{1+p} - \frac{1}{2+p} = \frac{1}{4n^2+6n+2},$$

which implies that $\omega + \varepsilon = \frac{1}{p+1}$. Thus, it is easy to see that $n\varepsilon < \omega$, i.e.,

$$\varepsilon < \frac{\omega}{n} < 1. \tag{3}$$

If $\text{opt}_{\text{SIM}}(f(\phi))$ denotes the value of an optimal solution for FFDCJ-SIMILARITY with instance $f(\phi)$ and c^* denotes the number of helpful cycles in an optimal solution of FFDCJ-SIMILARITY, then we have immediately that

$$\frac{\text{opt}_{\text{SIM}}(f(\phi))}{\omega+\varepsilon} \leq c^* \leq \frac{\text{opt}_{\text{SIM}}(f(\phi))}{\omega}. \tag{4}$$

Besides that

$$0 \leq c^* \leq n, \tag{5}$$

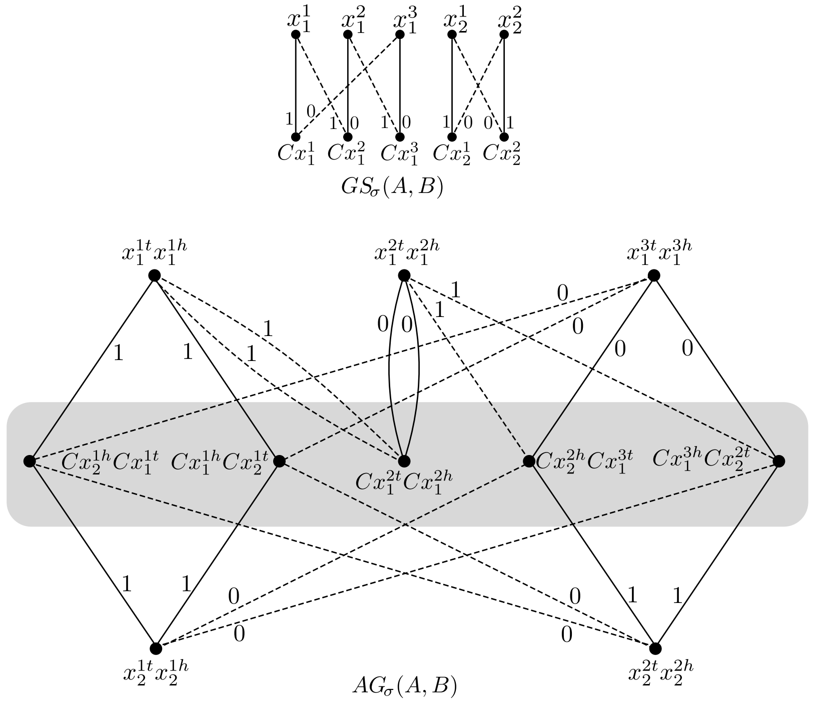

Figure 1 $GS_\sigma(A,B)$ and $AG_\sigma(A,B)$ for genomes $A = \{(x_1^1),(x_1^2),(x_1^3),(x_2^1),(x_2^2)\}$ and $B = \{(Cx_1^1\ Cx_2^1),(Cx_1^2),(Cx_1^3\ Cx_2^2)\}$ given by function f (Theorem 1) applied to $2\text{SAT}(3)$ clauses $C_1 = (x_1 \vee x_2)$, $C_2 = (\overline{x_1})$ and $C_3 = (\overline{x_1} \vee x_2)$. In $GS_\sigma(A,B)$, solid edges correspond to ex_i^j and dashed edges correspond to $e\overline{x_i}^j$. In $AG_\sigma(A,B)$, shaded region corresponds to genes of genome B, and solid (dashed) edges correspond to solid (dashed) edges of $GS_\sigma(A,B)$.

and

$$c^*\omega \leq \text{opt}_{\text{SIM}}(f(\phi)) \leq c^*(\omega+\varepsilon). \tag{6}$$

Thus, we have

$$\begin{aligned} c^*(\omega+\varepsilon) &= c^*\omega + c^*\varepsilon \\ &< c^*\omega + \frac{c^*\omega}{n} \quad (7)\\ &\leq c^*\omega + 1\cdot\omega \quad (8)\\ &= c^*\omega + \omega, \quad (9)\end{aligned}$$

where (7) comes from (3) and (8) is valid due to (5).

Now, let c^r be the number of helpful cycles given by an approximate solution for the FFDCJ-SIMILARITY with approximation ratio r. Then,

$$R_{\text{MAX-2SAT3}}(\phi, g(f(\phi),\gamma)) = \frac{\text{opt}_{\text{2SAT3}}(\phi)}{c^r} = \frac{c'}{c^r} \leq r,$$

where the last inequality is given by Proposition 3 below. This concludes the first part of the proof. □

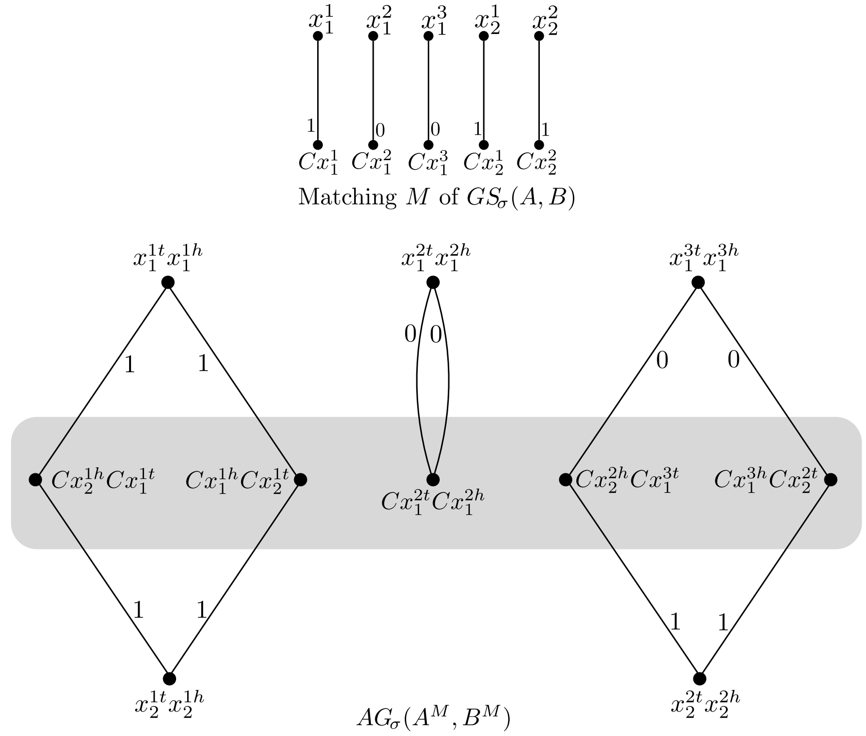

Figure 2 A matching M of $GS_\sigma(A, B)$ and cycles induced by M in $AG_\sigma(A^M, B^M)$ for genomes of Fig. 1. This solution of FFDCJ-SIMILARITY represents clauses C_1 and C_3 of MAX-2SAT3 satisfied.

Proposition 2 *Let c' be the number of helpful cycles in a feasible solution of* FFDCJ-SIMILARITY *with the greatest number of helpful cycles possible. Let c^* be the number of helpful cycles in an optimal solution of* FFDCJ-SIMILARITY. *Then,*

$$c' = c^*.$$

Proof. Since c' is the greatest number of helpful cycles possible, it is immediate that $c^* \le c'$.

Let us now show that $c^* \ge c'$. Suppose for a moment that $c^* < c'$. Since c^* and c' are integers, this implies that $c^* + 1 \le c'$, i.e.,

$$c^* \le c' - 1. \tag{10}$$

Let $\mathcal{C}'$ be the set of cycles with c' cycles, i.e., with the maximum number of helpful cycles possible. Let $\widehat{w}(\mathcal{C}') := \sum_{C \in \mathcal{C}'} \widehat{w}(C) = \sum_{C \in \mathcal{C}'} w(C)/|C|$. Then

$$\begin{aligned}
\widehat{w}(\mathcal{C}') \ge c'\omega &= (c' - 1)\omega + \omega \\
&\ge c^*\omega + \omega \qquad (11)\\
&> c^*(\omega + \varepsilon) \qquad (12)\\
&\ge \mathrm{opt}_{\mathrm{SIM}}(f(\phi)), \qquad (13)
\end{aligned}$$

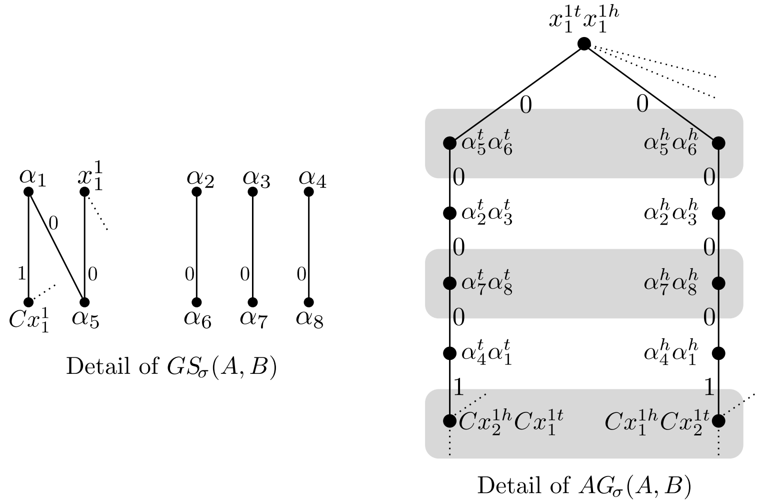

Figure 3 Detail of graphs $GS_\sigma(A,B)$ and $AG_\sigma(A,B)$ for genomes of Fig. 1 including extenders for edge (x_1^1, Cx_1^1) for $p=4$. Shaded regions correspond to genes of genome B. Extending all edges of weight 1 and selecting the matching of Fig. 2, this helpful cycle (only half of it is in this figure) would have normalized weight $\frac{4}{4(p+1)} = \frac{1}{p+1} = \frac{1}{5} = 0.2$.

where (11) follows from (10), (12) comes from (9), and (13) is valid due to (6). It means that $\widehat{w}(\mathcal{C}') > \text{opt}_{\text{SIM}}(f(\phi))$, which is a contradiction.

Therefore, $c' = c^*$. □

Proposition 3 *Let c^r be the number of helpful cycles given by an approximate solution for* FFDCJ-SIMILARITY *with approximation ratio r. Let c' be the same as defined in Proposition 2. Then,*

$$c^r \geq \frac{c'}{r}.$$

Proof. Given an instance $f(\phi)$ of FFDCJ-SIMILARITY, let γ^r be an approximate solution of $f(\phi)$ with performance ratio r, i.e., $\text{val}(f(\phi), \gamma^r) \geq \frac{\text{opt}_{\text{SIM}}(f(\phi))}{r}$. Let c^r be the number of helpful cycles of γ^r. Then

$$\begin{aligned}
c^r &\geq \frac{\left(\frac{\text{opt}_{\text{SIM}}(f(\phi))}{r}\right)}{\omega+\epsilon} \\
&> \frac{\text{opt}_{\text{SIM}}(f(\phi))}{r(\omega+\omega/n)} && (14)\\
&= \frac{\text{opt}_{\text{SIM}}(f(\phi))}{r\omega} \cdot \frac{n}{n+1} \\
&\geq \frac{c'\omega}{r\omega} \cdot \frac{n}{n+1} && (15)\\
&= \frac{c'}{r} \cdot \left(1 - \frac{1}{n+1}\right) \\
&= \frac{c'}{r} - \frac{c'}{r(n+1)} \\
&\geq \frac{c'}{r} - 1, && (16)
\end{aligned}$$

where (14) follows from (3), (15) is valid from (6) and Proposition 2. Then, from (16) we know that $c^r > \frac{c'}{r} - 1$ and, since c^r is an integer number, the result follows. □

We now continue with the proof of Theorem 1.

Proof.[Theorem 1, second part] First, notice that if a problem is APX-hard, the existence of a PTAS for it implies P = NP. Since a strict reduction preserves membership in the class PTAS, finding a PTAS for FFDCJ-SIMILARITY implies a PTAS for every APX-hard problem and P = NP. A PTAS for FFDCJ-SIMILARITY would also imply an approximation ratio better than $2012/2011 = 1.0005\ldots$, unless P = NP. This follows immediately from the reduction in Theorem 1 with $R_{\text{MAX-2SAT3}} = R_{\text{FFDCJ-SIMILARITY}}$ and the fact that MAX-2SAT3 is shown in [2] to be NP-hard to approximate within a factor of $2012/2011 - \varepsilon$ for any $\varepsilon > 0$.

However, our result is slightly stronger. Notice particularly that the reduction MAX-2SAT3 $\leq$ FFDCJ-SIMILARITY from the first part of the proof can be trivially extended to MAX-2SAT $\leq$ FFDCJ-SIMILARITY by extending variable components to arbitrary sizes. This increases the lower bound to $22/21 = 1.0476\ldots$ [5]. □

References

1. Crescenzi, P.: A short guide to approximation preserving reductions. In: Proceedings of Computational Complexity. Twelfth Annual IEEE Conference, pp. 262–273 (1997)
2. Berman, P., Karpinski, M.: On some tighter inapproximability results. In: Proc. of ICALP 1999, pp. 200–209 (1999). Springer
3. Ausiello, G., Protasi, M., Marchetti-Spaccamela, A., Gambosi, G., Crescenzi, P., Kann, V.: Complexity and Approximation: Combinatorial Optimization Problems and Their Approximability Properties. Springer, Heidelberg (1999)
4. Raman, V., Ravikumar, B., Rao, S.S.: A simplified np-complete maxsat problem. Information Processing Letters **65**(1), 1–6 (1998)
5. Håstad, J.: Some optimal inapproximability results. Journal of the ACM (JACM) **48**(4), 798–859 (2001)
